# Recurrent Thyrotoxic Periodic Paralysis As the Sole Clinical Manifestation of Untreated Hyperthyroidism

**DOI:** 10.7759/cureus.53581

**Published:** 2024-02-04

**Authors:** Zhanna Zavgorodneva, Muzammil Khan

**Affiliations:** 1 Department of Internal Medicine, Brookdale University Hospital Medical Center, Department of Internal Medicine, One Brooklyn Health Systems, New York, USA; 2 Department of Endocrinology, Diabetes and Metabolism, SUNY (State University of New York) Downstate Health Science University, New York, USA

**Keywords:** graves disease, thyrotoxicosis, muscle paralysis, hypokalemia, thyrotoxic periodic paralysis, hyperthyroidism

## Abstract

Thyrotoxic periodic paralysis (TPP) is a complication of hyperthyroidism that predominantly affects the Asian population. Episodes of muscle paralysis typically coincide with symptoms of hyperthyroidism. However, we present a unique case of TPP in a 32-year-old African American patient where TPP served as the primary manifestation of thyrotoxicosis. The patient was discharged with a resolution of symptoms after correcting electrolyte abnormalities.

## Introduction

Thyrotoxic periodic paralysis (TPP) is a rare yet severe complication of hyperthyroidism, marked by episodes of muscle paralysis and hypokalemia in the presence of a hyperthyroid state. Although TPP traditionally primarily affects the Asian population [1], this condition is increasingly spreading among different ethnic groups due to global migration and intermarriage. Healthcare professionals from diverse countries must be prepared to recognize and treat this condition. In this presentation, we share a case of TPP, shedding light on its epidemiological and clinical challenges.

## Case presentation

A 32-year-old African American male presented to the hospital with complaints of severe generalized weakness, characterized by a sudden onset of insufficient strength to stand from the bed and take a few steps. The patient reported having a substantial dinner and consuming two shots of alcohol on the night before the onset of symptoms. Apart from subjective weakness, there were no symptoms of hypothyroidism. During the initial episode in 2022, the patient was diagnosed with hyperthyroidism, and the attack was considered a manifestation of hyperthyroidism itself. The patient received consultation from an endocrinologist, who prescribed thionamide antithyroid medication. However, the patient reported non-adherence to the treatment plan, with self-reported periods of up to six months without any medications.

**Figure 1:**
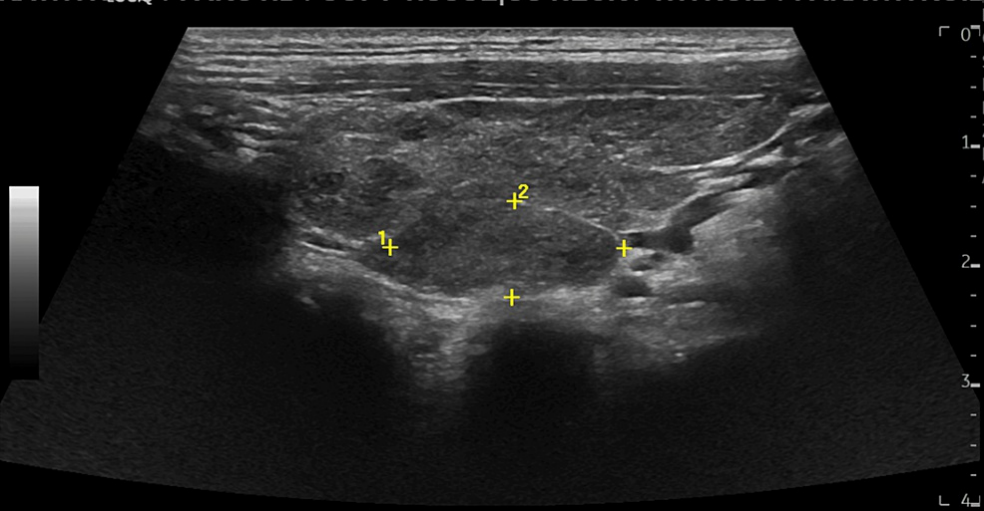
Right thyroid nodule A 2.2 x 1.0 cm hypoechoic homogenous nodule with well-defined borders was identified on ultrasound examination of the neck.

**Figure 2:**
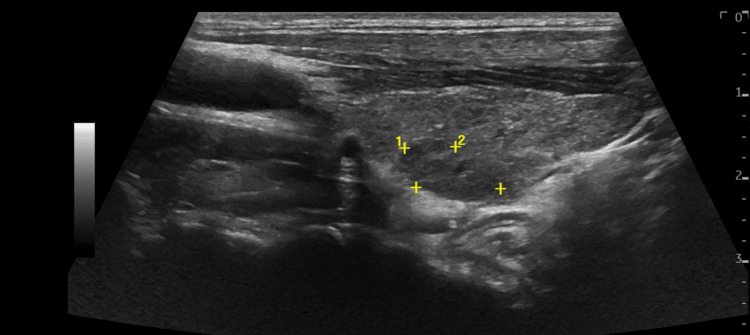
Left thyroid nodule A 1.3 x 0.8 cm hypoechoic homogenous nodule with well-defined borders was identified on ultrasound examination of the neck.

Physical examination revealed proximal muscle weakness in both legs, decreased knee reflex bilaterally, and no other neurological deficits. Vital signs were within normal ranges. Laboratory findings showed potassium (K) at 2.7 mmol/L (normal range 3.4-5.1 mmol/L), magnesium (Mg) at 1.54 mg/dL (normal range 1.8 to 2.2 mg/dL), phosphorus (Ph) at 1.7 mg/dL (normal range 2.8 to 4.5 mg/dL), thyroid-simulating hormone (TSH) <0.010 uIU/mL (normal range 0.4 to 4.5 uIU/mL), and free T4 at 2.56 ng/dL (normal range 0.9 to 1.8 ng/dL). Thyroid antibodies were checked, revealing thyroid peroxide antibody levels at 323 IU/mL (normal range < 9 IU/mL), thyroid-stimulating immunoglobulin 2.43 IU/L (normal range < 0.55 IU/L), and thyrotropin receptor antibodies 2.03 IU/L (normal range < 1.58 IU/L). The EKG showed a prolonged QT segment and U wave. Thyroid ultrasound revealed a markedly heterogeneous enlarged thyroid gland and bilateral thyroid nodules measuring 2.2 cm and 1.3 cm in their maximum dimensions (Figure 1, Figure 2).

The patient reported several similar episodes occurring over the last two years following the initial diagnosis of hyperthyroidism. All these episodes were associated with hypokalemia and worsening of hyperthyroidism after discontinuation of treatment by the patient (Table 1). Although the patient was referred for a nuclear thyroid test to exclude overlapping hot nodules, he refused to proceed with this test. His family history was significant for hyperthyroidism in his mother.

**Table 1:** Dynamics of thyroid function tests and electrolytes The patient had three hospitalizations due to TPP with acute symmetrical lower limb weakness, hypokalemia, and evidence of a hyperthyroid state. TPP: thyrotoxic periodic paralysis; TSH: thyroid-stimulating hormone

Date/parameters	K (mmol/L)	free T4 (ng/dL)	TSH (uIU/mL	Mg (mg/dL)
6/23/16	3.7			
10/26/2022 - admission #1	3.3	2.36	0.015	1.5
10/30/22	4.5	1.58		
3/28/2023 - admission #2	2.3	2.41	0	1.6
4/3/23	4.8	1.97		
10/17/2023 - admission #3	2.7	2.56	0.01	1.54
10/19/23	4.1			

The patient was diagnosed with thyrotoxic periodic paralysis. The mainstay treatment of this condition is the correction of electrolyte abnormalities and addressing hyperthyroidism. Potassium replacement was provided by the intravenous and oral routes with a daily dosage of 80-120 mEq a day and magnesium replacement was by intravenous route with 2-3 g a day. Methimazole was started in the dosage of 5 mg every 8 hours according to Americal Thyroid Association guidelines. After electrolyte replacement and reinitiation of thionamide, the patient experienced improvement and was discharged two days after admission, when potassium replacement was not required anymore. Instructions were provided regarding the importance of medication compliance and follow-up. Additionally, the patient was advised to avoid high-carbohydrate meals and strenuous physical exercise to prevent the recurrence of his condition.

## Discussion

The repeated episodes of proximal muscle weakness, accompanied by hypokalemia and biochemical hyperthyroidism, clearly indicate the diagnosis of TPP. The overall presentation aligns with the typical manifestations of this condition, including a precipitating event, such as a high-carbohydrate meal, an abrupt onset of symptoms upon awakening, a history of similar attacks, and the reversibility of the attack once hypokalemia and hyperthyroidism are addressed. Notably, the case is unique due to the patient's ethnic background and the subtlety of hyperthyroidism signs compared to neurological manifestations.

Several studies have delved into the mechanisms of TPP. Chan et al. demonstrated increased Na-K-ATPase activity in thyrotoxic subjects with and without paralysis [2]. Similarly, Kjeldsen et al. found an increase in 3H-ouabain binding sites (a molecular indicator of ATPase activity) in the skeletal muscles of patients with hyperthyroidism [3]. This could potentially explain the mechanism behind the precipitation of TPP after consuming a high-carbohydrate meal, followed by hyperinsulinemia and intracellular potassium shifting. Intracellular potassium shifting and the onset of TPP can also be triggered by factors such as stress, certain drugs, and intense physical exercise. Another mechanism involves increased β-adrenergic activity in a hyperthyroid state, amplifying the production of intracellular cAMP. Additionally, alterations in K+ efflux play a role in TPP [4]. Genetic studies have revealed various gain-of-function and loss-of-function mutations associated with periodic paralysis [5,6].

Periodic thyrotoxic paralysis is considered a rare complication of hyperthyroidism. A study from Mayo Clinic reported 10 cases over 20 years, with a prevalence of 0.1-0.2% in thyrotoxic patients in North America [7]. While the condition predominantly presents in Chinese men [1], it has been reported in diverse ethnic groups, including Hispanic, Native American, Caucasian, Afro-Caribbean, Afro-Trinidadian, and African populations [5,8-12].

In this case, the patient, diagnosed with hyperthyroidism in 2022, never developed signs of thyrotoxicosis. Instead, periodic paralysis itself represented the clinical features of the patient's hyperthyroid state. This contrasts with previous findings, as the majority of patients with TPP usually experience thyrotoxic symptoms [13]. The patient's persistent hyperthyroid state over two years, due to nonadherence to treatment, did not lead to thyrotoxicosis or thyroid storm but resulted in multiple admissions for periodic thyrotoxic paralysis. During the current admission, a relapse of paralysis was provoked by a high-carbohydrate meal, though it could also follow heavy exercise or acute illness. Hypokalemia, a hallmark of this condition, can be life-threatening [14]. Correction of hypokalemia, along with β-blockers and anti-thyroid medications, constitutes the mainstay of treatment for TPP. The absence of other symptoms, coupled with repeated episodes of weakness and significantly low potassium levels, renders the patient vulnerable to life-threatening arrhythmias and sudden death.

## Conclusions

Thyrotoxic periodic paralysis is a rare complication of hyperthyroidism that may go unrecognized, especially in patients with an ethnicity unusual for this condition and when it serves as the sole manifestation of a hyperthyroid state. It is advisable to consider routine thyroid function tests for patients experiencing recurrent episodes of paralysis with concurrent hypokalemia.

## References

[1] McFadzean AJ, Yeung R (1967). Periodic paralysis complicating thyrotoxicosis in Chinese. Br Med J.

[2] Chan A, Shinde R, Chow CC, Cockram CS, Swaminathan R (1991). In vivo and in vitro sodium pump activity in subjects with thyrotoxic periodic paralysis. BMJ.

[3] Kjeldsen K, Nørgaard A, Gøtzsche CO, Thomassen A, Clausen T (1984). Effect of thyroid function on number of Na-K pumps in human skeletal muscle. Lancet.

[4] Lin SH, Huang CL (2012). Mechanism of thyrotoxic periodic paralysis. J Am Soc Nephrol.

[5] Glass J, Osipoff J (2020). Thyrotoxic periodic paralysis presenting in an African-American teenage male: case report. Int J Pediatr Endocrinol.

[6] Ryan DP, da Silva MR, Soong TW (2010). Mutations in potassium channel Kir2.6 cause susceptibility to thyrotoxic hypokalemic periodic paralysis. Cell.

[7] Kelley DE, Gharib H, Kennedy FP, Duda RJ Jr McManis PG (1989). Thyrotoxic periodic paralysis. Report of 10 cases and review of electromyographic findings. Arch Intern Med.

[8] Satoyoshi E, Murakami K, Kowa H (1963). Periodic paralysis in hyperthyroidism. Neurology.

[9] Ober KP (1992). Thyrotoxic periodic paralysis in the United States. Report of 7 cases and review of the literature. Medicine (Baltimore).

[10] M Ramdath, J Teelucksingh, A Ramnath (2017). Thyrotoxic hypokalaemic periodic paralysis: a case series from Trinidad, West Indies. West Indian Med J.

[11] Sow M, Diagne N, Djiba B (2020). Thyrotoxic hypokalemic periodic paralysis in two African black women [Article in French]. Pan Afr Med J.

[12] Boissier E, Georgin-Lavialle S, Cochereau D, Ducloux R, Ranque B, Aslangul E, Pouchot J (2013). Thyrotoxic periodic paralysis: a case series of four patients and literature review [Article in French]. Rev Med Interne.

[13] Ko GT, Chow CC, Yeung VT, Chan HH, Li JK, Cockram CS (1996). Thyrotoxic periodic paralysis in a Chinese population. QJM.

[14] Wu CZ, Wu YK, Lin JD, Kuo SW (2008). Thyrotoxic periodic paralysis complicated by acute hypercapnic respiratory failure and ventricular tachycardia. Thyroid.

